# Skin Diseases among the Old Age Residents in a Nursing Home: A Neglected Problem

**DOI:** 10.1155/2020/8849355

**Published:** 2020-11-05

**Authors:** Abbas Darjani, Narges Alizadeh, Elahe Rafiei, Meysam Moulaei, Seyed Hamed Naseri Alavi, Hojat Eftekhari, Rana Rafiei, Kaveh Gharaei-Nejad, Zahra Mohtasham-Amiri

**Affiliations:** ^1^Department of Dermatology, Guilan University of Medical Sciences, Rasht, Iran; ^2^Guilan University of Medical Sciences, Rasht, Iran; ^3^Preventive and Community Department, Guilan University of Medical Sciences, Rasht, Iran

## Abstract

**Background:**

Geriatric health care has become a worldwide concern, but a few statistical studies were carried out about skin diseases in this age group in the nursing home of Iran.

**Aims:**

In this study, we set out to determine the frequency as well as the age and gender distribution of dermatological diseases in nursing home old age residents.

**Methods:**

In a cross-sectional study, all patients over 60 years who were living in a charity nursing home complex of Rasht in 2017 participated in this study. Baseline information on sociodemographic variables, past medical history, and medication were gathered by medical staff during a face-to-face interview. Full-body skin examination was done by dermatologists. Biopsy, and pathological and laboratory methods were used to confirm the diagnosis of suspected lesions or disease.

**Results:**

In this study, 259 people underwent the study. 52.9% were male, and their mean age was 73.5 years (SD = 9.1 years). Hypertension (20.9%); diabetes mellitus (9.7%), and hypothyroidism (2.3%) were the most common underlying diseases. Most of them (85.7%) had age-related skin changes. The benign neoplasm was the most common skin disease among patients (68.3%), followed by infectious diseases (46.3%) and erythemo-squamous (31.6%). None of them had precancerous lesions or skin cancers. There were not any differences between skin disorders and gender or age groups in this study.

**Conclusion:**

Our study suggests that skin manifestations and diseases are common among nursing home old age residents in this area. Therefore, this should constitute one of the top priorities of aged care physicians and nurses.

## 1. Introduction

Improvement in health care and control of chronic diseases have increased lifespan and prompted rapid growth of the elderly population [1]. Iran has started to come across the population ageing phenomenon too. The proportion of the elderly is projected to double in less than 20 years. While the proportion of people with 60 years old age and above in Iran was 5.4%in 1975; it will increase to 10.5% in 2025 and 21.7% in 2050 [2, 3]. According to the Census of Population and Housing 2016 of Iran, Guilan Province in the north of Iran with 11.9% population more than 60 years has the highest elderly rate in Iran [4].

The ageing process is determined by numerous intrinsic and extrinsic factors and affects all organs and tissues, including the skin. In ageing, a decline in the regular functions of the skin is observed. As a result, some inevitable changes, such as roughness, wrinkling, and laxity of the skin, and atypical presentations of dermatologic diseases are observed in elderly patients [5].

Skin ageing, functional limitations, chronic diseases, polypharmacy, personal skin care, and hygiene habits in populations aged ≥65 years cause increased vulnerability to skin diseases and cutaneous problems [6]. Many previous studies showed a high prevalence of skin disorders in old age residents [7–9], but most of the published articles were obtained in hospital or outpatient clinic settings [10–12]. There is the limited literature on skin diseases in nursing homes [13–15]. The staff of these centers are more likely to pay attention to bed sores or incontinence-associated dermatitis [6]. Therefore, cutaneous diseases in institutional long-term care settings are largely unknown. We conducted the first study about skin diseases in nursing home patients within the fast-growing region of Iran.

## 2. Methods and Material

This study was carried out in a large charity nursing home complex with a history of more than 60 years in Guilan, north of Iran. After coordination with the director of the center, in a cross-sectional study, all of the residents more than 60 years participated in this study from Nov 2017 to March 2018. Informed consent was received from the patients or their care-givers. Baseline information on sociodemographic variables, past medical history, and medication were all gathered by medical staff during a face-to-face interview. Also, their medical records were reviewed carefully. Full-body skin examination was done by dermatologists. Biopsy, and pathological and laboratory methods were used to confirm the diagnosis of suspected lesions or disease. Physicians' visits and any procedures for diagnosis and treatments were free of charge. The diseases were categorized into seven different groups including erythemo-squamous diseases (such as psoriasis, lichen planus, seborrheic dermatitis, contact dermatitis, Paederus dermatitis, stasis dermatitis, lichen simplex chronics, and pilaris rubra pityriasis), infectious diseases (fungal, bacterial, and viral infections and infestations), benign neoplasm (pillar cyst, keloids, lipoma, seborrheic keratosis, pyogenic granuloma, epidermal cyst, keratoacantom, and skin tag), precancerous lesions (leukoplakia, actinic keratosis, and Bowen), skin cancer (basal cell carcinoma (BCC), squamous cell carcinoma (SCC), mycosis fungoides, and Kaposi's sarcoma), age-related skin changes (xerosis, senile lentigo, senile comedon, angioma, and nail ridging), and the others (leg ulcer, insect bite, sarcoidosis, ingrowing toe nail, corn, vasculitis, and vitiligo). In case of any suspicion of the disease, case presentation between dermatologists was performed. Follow-up after treatment of infectious diseases was done. Data analysis was carried out with the SPSS Software, version 18. Descriptive statistics for the prevalence of skin disease were calculated, and gender difference was investigated using chi square testing. In all the analyses, a *p* value of <0.05 was considered statistically significant.

## 3. Results

In this study, 259 eligible residents of the nursing home were recruited. As shown in Table 1, 52.9%were male, with a mean age of 73.5 years (SD = 9.1 years) and a range of 60 to 96 years. Most participants were in the age group of 60–69 years (39.8%), and 106 persons (40.9%) were illiterate. The previous jobs of men were farmer (41%), self-employment (34.3%), and retired (13%), respectively.

Interestingly, 157 persons (60.6) did not have any underlying diseases, among others, HTN (20.9%); DM (9.7%) and hypothyroidism (2.3%) were the most common underlying diseases, respectively. All patients with at least one underlying disease have received more than three medications. Only 7 patients (2.3%) were bedridden.

Most of them (85.7%) had age-related skin changes. The benign neoplasm was a common skin disease among patients (68.3%), followed by infectious diseases (46.3%) and erythemo-squamous (31.6%). Most of the participants had more than one skin disease. No precancerous lesions or skin cancers were seen.

The most prevalent erythemo-squamous diseases were seborrheic dermatitis (15.4%), psoriasis (7.3%), and lichen simplex (3.5%). Fungal infections (tinea and candidiasis) were the most common infectious diseases (37.8%) as followed by viral infections (11.2%) and infestations (scabies) (4.3%). The most common age-related skin changes were increased, longitudinal nail lines (49.6%), cherry angioma (46.3%), and lentigo (35.9%). Seborrheic keratosis (49.4%) and skin tag (27.4%) were the most common benign neoplasm. Eczema (37.5%) and nevus (13.9%) were common problems in other dermatological diseases. There were not any differences between skin disorders and gender or age groups in this study (Tables 2 and 3).

## 4. Discussion

In parallel to the increasing population of old people and change of lifestyle, this age group requires more care which leads an increasing number of residents in the nursing homes. Therefore, understanding the unmet needs and common diseases among them is very important.

There was no significant difference in skin diseases between the sexes and ages. Most of them were in the 60–69 age group. Indeed, most of the residents of the nursing home in this study were young-old age. In other studies, most old age residents were female and older ages [6, 7, 13–19]. These differences may partly explain the difference between skin lesions in this study and previous studies.

In our study, the benign neoplasm group was the most common skin disease present among the participants after age-related skin changes, affecting more than two-thirds of them (68.3%). Seborrheic keratosis (49.4%) was the most common benign neoplasm. This rate is different from other studies on elderly populations, where prevalence of 1.7% to 85% has been recorded [5, 19–23] and inconsistent with the previous study in this geographic area [12]. Seborrheic keratosis is the most common benign epithelial tumor of adulthood. These differences may be related to different settings of participants (population-based, clinic-based, or nursing home), and different climate and jobs of participants. It seems that sunlight exposure may play a role because seborrheic keratoses are common on sun exposed areas such as the back, arms, face, and neck [24]. The majority of patients were exposed to sunlight due to their jobs.

More than one-fourth of patients had skin tags (27.4%). Obesity and overweight (even temporary increases in weight) dramatically increase the chances of having skin tags [25]. Unfortunately, we did not record the weight and height of patients to reveal the relation between skin tag and obesity in our patients. It is recommended that this relationship be investigated in future studies.

Infectious diseases (46.3%) were the second skin disease in the patients that was in consistent with other studies [26–30]. Fungal infections were the most common infectious diseases in our study that were found in 37.8%. Other studies showed different rates from 4.4% to 61.6% [13, 18, 21, 22, 27–29]. Fungal infection depends on underlying diseases such as diabetes, bedridden status, and also hygiene level of patients. It seems that infectious diseases are more prevalent among nursing home care patients in comparison to outpatients' clinic patients [8, 12].

Unlike other studies, no precancerous lesions or skin cancers were seen in this study [12, 19, 21, 23, 31]. One study on 398 elderly people staying in nursing homes in Taiwan has reported skin cancer in only one case [17]. We suspect that age was probably an important factor in this difference, as the average age of our patients (73.5 years) was much lower than in the previous study. Perhaps the reason for this finding is the presence of a general physician in the nursing home who is responsible for screening and referring patients suspected of having malignancies to the outpatient dermatological clinics. Another reason may be the skin type of these people, and most of them had type III skin.

Eczema was another common problem in this study that affected nearly half of the patients. A previous study in this area showed a lower rate of 16% [12]. In many previous studies, eczema was the most frequent skin problem in the elderly [32–34], but some studies reported a low rate of this problem [13, 35, 36]. Dry skin and eczema in older people are often due to physiological ageing. Many intrinsic factors in older people such as general health changes/chronic illness (diabetes, renal failure, thyroid disease, iron deficiency, or jaundice), poor dietary and fluid intake, medications, allergies, physical limitations, urinary/faecal incontinence (skin maceration and friction caused by absorbent pads) mental state, and personal hygiene in addition to environmental factors (extrinsic) including overheating (central heating and sitting by fires), low air humidity of the atmosphere, overwashing with soaps and detergents, seasonal changes (cold winters/hot summers), and household/garden irritants and allergies affect the skin and may help trigger an eczema flare-up [37]. Due to the wide causes of eczema, its wide range is expected in the elderly.

## 5. Limitation

This was a cross-sectional study with its potential limitation. On the other hand, this study was carried out in only one charity center and the socioeconomic situation of people who live here was different from the community-dwelling old age residents, so these patients may not be a representative sample of all old age residents in this area. Also, background diseases were not specified and laboratory data were not available in many patients' records.

## 6. Conclusions and Implications

Our study suggests that skin manifestations and diseases are common among nursing home patients in this area of Iran. Also, we have a documented picture of skin diseases in this ageing group that can be used for education and development of skin care policy by aged care physicians and nurses.

## Figures and Tables

**Table 1:** Patient demographic characteristics.

Variable	Number	Percent
*Age group (year)*		
60–69	103	39.8
70–79	80	30.9
≥80	76	29.3

*Gender*		
Male	137	52.9
Female	122	47.1

*Education*		
Illiterate	106	40.9
Primary school	74	28.6
High school	64	24.7
Academic degree	15	5.8

*Occupation*		
Housewife	100	38.6
Farmer	56	21.6
Self-employment	47	18.5
Retired	29	11.2
Other	27	10.1

**Table 2:** The distributions of all skin diseases according to gender.

Disease							
Gender	Total no. (259)		Male no. (137)		Female no. (122)		*p* value
	No.	%	No.	%	No.	%	
Erythemo-squamous diseases	82	31.7	43	31.4	39	32	NS
Infectious diseases	120	46.3	59	43.1	61	50	NS
Benign neoplasm	177	68.3	95	69.3	82	67.2	NS
Age-related skin changes	222	85.7	118	86.1	104	85.3	NS
Others	164	63.3	82	59.9	21	67.2	NS

**Table 3:** The most prevalent skin diseases according to age groups.

Disease							
Age groups	60–69 years no. (103)		70–79 years no. (80)		≥80 years no. (76)		*p* value
	No.	%	No.	%	No.	%	
Erythemo-squamous diseases	31	30.1	28	35	23	30.3	NS
Infectious diseases	46	44.7	36	45	38	50	NS
Benign neoplasm	66	64.1	60	75	51	67.1	NS
Age-related skin changes	89	86.4	68	85	65	85.5	NS
Others	64	62.1	48	60	52	68.4	NS

## Data Availability

The study protocol and the datasets analyzed are available from the corresponding author upon request.
